# Steatocystoma simplex of the lacrimal caruncle: a case report

**DOI:** 10.1186/s12886-016-0358-2

**Published:** 2016-10-19

**Authors:** Yuichiro Ishida, Yasuhiro Takahashi, Emiko Takahashi, Yoshiyuki Kitaguchi, Hirohiko Kakizaki

**Affiliations:** 1 Department of Oculoplastic, Orbital & Lacrimal Surgery, Orbital & Lacrimal Surgery, Aichi Medical University Hospital, 1-1 Yazako-Karimata, Nagakute, Aichi 480-1195 Japan; 2 Department of Pathology, Aichi Medical University Hospital, 1-1 Yazako-Karimata, Nagakute, Aichi 480-1195 Japan

**Keywords:** Steatocystoma, Lacrimal caruncle, Pathological findings

## Abstract

**Background:**

This is the third reported case of a steatocystoma simplex in the lacrimal caruncle.

**Case presentation:**

A 60-year-old male presented with a history of a slowly progressing mass in the right lacrimal caruncle since several years before his initial visit. At the first examination, a yellowish, relatively smooth surface mass was observed in the right lacrimal caruncle. The caruncular mass was completely removed under local anesthesia. The pathological findings of this mass were consistent with a steatocystoma. At the 6-month follow-up, there was no sign of recurrence or development of the steatocystoma or any other masses.

**Conclusion:**

Although steatocystoma simplex rarely occurs in the lacrimal caruncle, it needs to be considered as a possible diagnosis for patients with a mass lesion in the caruncle.

## Background

Steatocystoma is a benign tumor thought to be a circumscribed malformation arising from the pilosebaceous duct junction [1]. Steatocystoma simplex is a noninheritable solitary counterpart of a steatocystoma multiplex [1–6]. Steatocystoma simplex commonly occurs in the skin of the forehead, nose, scalp, neck, axillae, chest, upper limbs, back, or legs [2–6]. In contrast, steatocystoma simplex in the periocular region is extremely rare [4, 5], with only two reported cases of a lacrimal caruncular lesion [2, 3].

In this case report, we present a case of a steatocystoma simplex in the lacrimal caruncle.

## Case presentation

This study was approved by the ethics committee of Aichi Medical University Hospital (No. 2015-H011) and adhered to the tenets of the 1964 Declaration of Helsinki. Written informed consent was obtained from the patient for publication of this case report and any accompanying images.

A 60-year-old male presented with a history of a slowly progressing mass in the right lacrimal caruncle since several years before his initial visit. There was no family history of any similar masses. The patient had no history of removal of any similar masses.

**Fig. 1:**
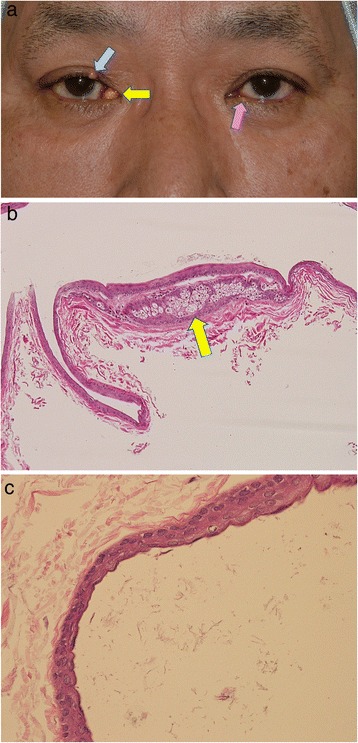
**a**. A photograph of the patient’s face. A yellow mass in the right caruncle (yellow arrow) and pale yellow nodules in the right upper (blue arrow) and left lower eyelid margins (pink arrow) are shown. **b & c**. Pathological findings. **b**. A cystic mass is lined with stratified squamous epithelium and the cyst wall contains the sebaceous gland (arrow) (hematoxylin & eosin stain; magnification × 100). **c**. The cyst wall is three to four cells thick and is undulated without a granular cell layer (hematoxylin & eosin stain; magnification × 400)

At the first examination, a yellowish, relatively smooth surface mass was observed in the right lacrimal caruncle (Fig. 1a). The patient also presented with similar masses in the right upper and left lower eyelid margins (Fig. 1a). Systemic examination did not detect any other skin lesions.

All three lesions were completely removed under local anesthesia without rupture. The cysts were not opened prior to histological processing. The pathological examination of the caruncular mass revealed a cyst lined by squamous epithelium (Fig. 1b). The cyst wall was thin and contained sebaceous glands (Fig. 1b and c) [5, 6]. Invaginations resembling hair follicles extended from the cyst wall into the surrounding stroma [7]. The epithelial layer was partially undulated (Fig. 1c) [5, 6]. Hairs were not found in the cyst lumen [5]. The findings were consistent with a steatocystoma. The pathological diagnoses of the other masses in the right upper and left lower eyelid margins were a hybrid epidermoid and apocrine cyst, and a hidrocystoma, respectively. At the 6-month follow-up, there was no sign of recurrence or development of the steatocystoma or any other masses.

## Discussion

We describe a case of a steatocystoma simplex in the lacrimal caruncle. Although 962 cases of lacrimal caruncular tumors have been previously reviewed in eight reports, none of them included steatocystomas [8–15]. Only two single case reports of a steatocystoma simplex in the lacrimal caruncle have been reported [2, 3].

The steatocystoma simplex is a cutaneous tumor thought to be a circumscribed malformation arising from the pilosebaceous duct junction [1]. As the lacrimal caruncle contains elements of both conjunctiva and skin [15], steatocystomas may have developed from the lacrimal caruncle, in a similar manner as other skin regions.

Differential diagnoses of the steatocystoma simplex in the lacrimal caruncle vary. An oncocytoma is a slowly progressing, cystic mass; however, it usually exhibits a reddish-blue tan color [10]. Although sebaceous gland hyperplasia, sebaceous gland adenoma, and lipogranuloma appear as yellow nodules, they present with different pathological findings [10]. Dermoid cysts show similar clinical and pathological findings to steatocystomas, although the dermoid cysts do not show an undulation in the epithelial layer without containing hairs in the lumen [5]. Cutaneous keratocysts also have a histopathological resemblance to steatocystoma by a festooned lining continuous with a thick stratified squamous wall that lacks a granular layer; however, sebaceous glands and lobules are typically lacking in the cutaneous cysts [5, 6].

## Conclusion

In conclusion, we present a rare case of a steatocystoma simplex in the lacrimal caruncle. Steatocystoma simplex therefore needs to be considered as a possible diagnosis for patients with a mass lesion in the caruncle.
